# Sensory Ecology of Water Detection by Bats: A Field Experiment

**DOI:** 10.1371/journal.pone.0048144

**Published:** 2012-10-25

**Authors:** Danilo Russo, Luca Cistrone, Gareth Jones

**Affiliations:** 1 Laboratorio di Ecologia Applicata, Dipartimento Ar.Bo.Pa.Ve., Facoltà di Agraria, Università degli Studi di Napoli Federico II Portici, Napoli, Italy; 2 School of Biological Sciences, University of Bristol, Bristol, United Kingdom; 3 Forestry and Conservation, Cassino, Italy; University of Western Ontario, Canada

## Abstract

Bats face a great risk of dehydration, so sensory mechanisms for water recognition are crucial for their survival. In the laboratory, bats recognized any smooth horizontal surface as water because these provide analogous reflections of echolocation calls. We tested whether bats also approach smooth horizontal surfaces other than water to drink in nature by partly covering watering troughs used by hundreds of bats with a Perspex layer mimicking water. We aimed 1) to confirm that under natural conditions too bats mistake any horizontal smooth surface for water by testing this on large numbers of individuals from a range of species and 2) to assess the occurrence of learning effects. Eleven bat species mistook Perspex for water relying chiefly on echoacoustic information. Using black instead of transparent Perspex did not deter bats from attempting to drink. In *Barbastella barbastellus* no echolocation differences occurred between bats approaching the water and the Perspex surfaces respectively, confirming that bats perceive water and Perspex to be acoustically similar. The drinking attempt rates at the fake surface were often lower than those recorded in the laboratory: bats then either left the site or moved to the control water surface. This suggests that bats modified their behaviour as soon as the lack of drinking reward had overridden the influence of echoacoustic information. Regardless of which of two adjoining surfaces was covered, bats preferentially approached and attempted to drink from the first surface encountered, probably because they followed a common route, involving spatial memory and perhaps social coordination. Overall, although acoustic recognition itself is stereotyped and its importance in the drinking process overwhelming, our findings point at the role of experience in increasing behavioural flexibility under natural conditions.

## Introduction

Because the risk of dehydration is the greatest physiological threat to life on land, drinking water is a fundamental resource for all terrestrial animals [1]. Due to their peculiar morphology and physiology, bats often face the risk of dehydration. Much water is lost through their body surface, especially via the respiratory system and the extensive surfaces of wing membranes [2], [3].

Although bats may show physiological adaptations to limit water loss, such as specific qualitative and quantitative chemical composition of the lipid matrix in the epidermis’ stratum corneum [4], compensating water loss by drinking is the main mechanism adopted by these mammals to counter dehydration. For this reason even hibernating bats may periodically arouse from torpor to drink [5], [6]. The importance of water availability has been emphasised in studies addressing the impact of climate change on bats [7] as well as in those modelling bat distribution patterns [8].

Water also represents a major source of minerals for bats: calcium – reproductive females need to restore the mineral reservoirs mobilized for skeletal development in pups [9], [10], [11]; or sodium, which is particularly limiting in tropical environments [12]. In some fruit-eating bats, minerals in water are important to counter the effects of secondary plant metabolites largely ingested at times of high energetic demand [13].

Sensory and behavioural adaptations to discover or localise water are therefore subject to strong selective pressure. A groundbreaking study [14] revealed that water recognition is innate and that bats use echoacoustic cues to locate smooth water surfaces. Accordingly, bats perceive any horizontal smooth surface as water because it provides a typical mirror-like reflection of echolocation calls. That study [14] was performed in the laboratory where this recognition process was recorded in 15 species from three families (Rhinolophidae, Vespertilionidae and Miniopteridae), i.e. the phenomenon seems taxonomically widespread among bats. The innate nature of water recognition also appears clear because naïve juveniles show it too [14].

Although it was also suggested [14] that other sensory cues may be integrated for the process of water recognition, at least in a laboratory setting these seemed to be of minor importance compared with echoacoustic cues. Behavioural studies in captivity offer unique chances of effectively controlling the experimental design and the influences of variables potentially affecting the results, but they also involve constraints [15] including the possible influence of an artificial environment, the limited number of individuals tested and the effects of stress on captive subjects. It is thus especially useful to validate the results obtained in captivity with experiments under natural conditions [16].

The main objective of our study was to build on previous laboratory work [14] to confirm its outcome under natural conditions, where we could test a large number of individuals from several species –11, six of which not studied previously [14]. We therefore hypothesise that bats will mistake artificial smooth horizontal surfaces for water in nature, and will attempt to drink from them.

Under natural conditions, where familiar cues and features of the drinking site occur, other sensory information as well as spatial memory may be involved to determine a precise “local” cognitive picture of water, so that any departure from the expected features could be noticed more readily than in a completely unfamiliar setting such as that of a laboratory. Although the echoacoustic recognition of water surface is innate [14], we hypothesise that especially at familiar drinking sites other sensory cues might play an important role. If so, bats should be less likely to be deceived by the artificial layer and more prone to detect and interpret environmental changes based on visual, olfactory, gustative or mechanoreceptorial cues, or spatial memory.

For one model species (*Barbastella barbastellus*) we also compared echolocation sequences emitted by bats approaching real and fake water respectively (an aspect not covered in the previous [14] laboratory study). We hypothesise there will be no difference in echolocation behaviour of bats approaching water and artificial smooth surfaces, providing further evidence that bats approaching fake water do actually try to drink rather than simply attempt to explore the artificial surface.

Besides, our experiment aimed to test the possible influence of learning under natural conditions and in a familiar area, where the location of other easily reachable water sources is known. We hypothesise that an unsuccessful drinking experience such as that determined by replacing real water with an artificial surface mimicking it would, after a few drinking attempts, override the influence of the acoustic water-like cues the latter provided. This would, in turn, prompt the bat to leave the site and move to the closest real water source available.

## Materials and Methods

### Experimental Design

Our experiments were performed in August 2011 at the Abruzzo Lazio and Molise National Park, in the Italian central Apennines, under permit from the Park’s authorities according to Law 6 December 1991 n° 394. The study involved no animal capture or handling so the permit only regarded observational work. For our experiment we selected three watering troughs designed to provide water for cattle and used by hundreds of drinking bats every night (D. Russo, *pers. obs.*). All sites were characterized by a similar surrounding habitat dominated by mature beech forest and pastures and were located at 1220–1563 m a.s.l.

For experiment 1, we used two structurally similar, adjoining watering troughs, ca. 6×1.5 m at each of two sites (sites A and B). In such cases we covered one trough with a 0.5 cm thick transparent Perspex sheet (treatment) whereas the other was left uncovered (control). The sheet was placed immediately above the real water surface. At the third site (site C) we used a single 12×1.5 m watering trough: in that case we laid a Perspex layer on half of it and left the remaining watering trough surface free. The transparent Perspex did not change the colour of the water surface but introduced potentially significant olfactory and tactile cues (detected by the bats which contacted the artificial surface when attempting to drink from it). We hypothesised that if the latter had no deterring effect on bats, they would show an equal likelihood of attempting to drink at either surface. To control for possible differences in bat use between the watering troughs, or their sections used as treatments, we repeated the experiment twice at each site, each time covering a different watering trough (or its section, as done for site C).Experiment 2 aimed to test the effect of colour. To test a larger number of bats we covered the watering trough (or a section of it for site C) where in experiment 1 we had recorded a higher drinking activity, this time using a black sheet of Perspex. Even in dim light the colour difference between the surface and the adjoining water was obvious to us and we assumed it to be clear to bats as some vespertilionids can discriminate small differences in brightness even at low light intensities [17]. Then we compared the levels of bat activity between the transparent (recorded in experiment 1) vs. black Perspex layers, assuming that if colour had no deterring effect on bats, they would show an equal likelihood of mistaking either black or transparent Perspex for water.

For both experiments, we preliminarily ensonified both the Perspex layer and water with a natural *Myotis mystacinus* call played back through a Pettersson D1000X detector and an L-400 loudspeaker (Pettersson Elektronik AB, Uppsala); the frequency range for the latter was 10–110 kHz. The speaker’s acoustic axis formed an angle of ca. 45° with the surface. Qualitative examination of waveforms and spectrograms of the echoes generated by the two surfaces recorded with another D1000X detector placed above the loudspeaker (sampling rate 384000 Hz) suggested there were no detectable difference in structure so we assumed the Perspex surfaces and the water to convey analogous echoacoustic information [14].

### Data Collection and Analysis

Bat activity was filmed continuously with a Sony Handycam HDR – XR520VE (focal distance 5.5–66.0 mm) nightshot videocamera mounted on a 1.8 m tripod positioned at least 2 m away from the watering trough to avoid interference with flying bats. The videocamera was located close to the watering trough’s major axis and oriented to include both the covered and uncovered water surfaces. In preliminary tests this setting proved most effective to distinguish the trajectories followed by approaching bats. The scene was illuminated with an additional infrared lamp. Bat echolocation calls were recorded with two Pettersson D1000X bat detectors which continuously sampled in the real-time mode (sampling rate 384000 Hz) and saved recordings onto 4 Gb flashcards. The bat detectors were placed on the edge of each watering trough (or its section) at ca. half of its length and the microphone directed toward its centre. Audio and video recordings were synchronized before starting the experiment so we could associate all filmed bats to their echolocation calls. When black Perspex was used (experiment 2), each minute we also recorded illuminance (in lux) at ground level with a Delta Ohm (Delta Ohm s.r.l., Padua, Italy) photo-radiometer (spectral range 450–760 nm, operational range 0–200,000 lux, resolution ≤200 lux = 0.1; >200 lux = 1). Each recording session started when the first bat approached the drinking site, generally within 20 min after sunset, and lasted 60 min. Temperature was similar across nights (ca. 16°C) and wind intensity was negligible so these were deemed to exert the same influence on bat activity across different trials.

To record the number of drinking attempts per bat, in the laboratory audio and video recordings were examined synchronously by two operators. In most cases this allowed us to keep track of the movement of all recorded bats and count repeated drinking attempt events. When a bat disappeared from both audio and video recordings it was assumed to have left. In a few such cases we also used the notes made in the field where we attempted to track visually a bat that had disappeared from the video screen to establish whether it had either left or moved to the control watering trough to drink. Although bats that left may have returned later to the site we assume this risk to be negligible at least for those that had drunk successfully. We also ideally divided the area around each watering trough in four quadrants and assigned the bats to one of them according to which quadrants they approached the trough from.

Sound analysis was used to identify bats to species. We used BatSound 4 (Pettersson Elektronik AB, Uppsala) to generate spectrograms with a 512·pt FFT Hamming window, 98% overlap (providing a 975 Hz frequency resolution). For bat identification, one call per sequence was selected at random among those with a good signal-to-noise ratio and measurements were taken according to [18].

For species identification we used simplified versions of the multivariate discriminant functions [18] developed respectively for species emitting frequency modulated calls (FM) and for those whose calls start with a broadband sweep and end with a narrowband tail (FM-QCF). Such functions only covered species representing >1% of bats mistnetted at the experiment sites in summers 2000–2011. The function for species emitting FM-QCF calls included Kuhl’s pipistrelle *Pipistrellus kuhlii* (probability of correct identification = 0.98), common pipistrelle *Pipistrellus pipistrellus* (0.98), Savi’s bat *Hypsugo savii* (1.00), Leisler’s bat *Nyctalus leisleri* (1.00) and Schreiber’s bat *Miniopterus schreibersii* (99.1) [Wilk’s λ = 0.01783, P<0.0001]. Probabilities of correct identification for species broadcasting FM calls were also high: greater mouse-eared bat *Myotis myotis* (0.81), whiskered bat *Myotis mystacinus* (0.69), Natterer’s bat *Myotis nattereri* (0.83), brown long-eared bat *Plecotus auritus* (0.96) and barbastelle bat *B. barbastellus* (0.90) [Wilk’s λ = 0.06734, P<0.0001]. Although *M. mystacinus* calls may be easily confused with those of *Myotis daubentonii* [18], [19], the latter was never mistnetted at any of the drinking sites used for the experiment during over 10 summers of bat surveys so in our sites the risk of misidentification was ruled out. While *M. mystacinus* is the most abundant bat in the beech forests where the experiments were carried out, *Myotis brandtii* and *Myotis alcathoe* – other possible sources of confusion – are only very rarely encountered there (D. Russo, *pers. obs.*). However, since those species were not covered by the classification function we cannot exclude that we misclassified few bats from those species as *M. mystacinus* but the effects on our analysis are certainly negligible. Other details, including sample sizes used to develop functions, are given in [18]. When possible, after a response was obtained the identification was improved further by looking at diagnostic features of the spectrogram. This was especially useful for *B. barbastellus* whose alternation of call types 1 ad 2 offers unambiguous species recognition [20].

For experiment 1 (transparent Perspex) the variables we tested were respectively the total number of approaches to either surface (Perspex vs. water), the number of individuals performing them, and the number of approaches per subject. We employed a repeated-measure General Linear Model (GLM) ANOVA (factors: site, watering trough covered during the experiment and surface type, i.e. Perspex or water) and entered site as a random factor. We first tested both the variables’ main effects and their interactions. We then removed the interactions when not significant. Only final models are presented here. When the residual distribution did not conform to normality according to a Ryan Joiner test, we log-transformed the data to meet the ANOVA assumptions.

For the experiment 2, for each site we first calculated the ratio between the numbers of passes (or bats) associated with either black or transparent Perspex and the corresponding total numbers of passes (or bats), i.e. those recorded over Perspex + those over water, observed during a trial. We used this ratio as a proxy for the number of times bats mistook Perspex for water. Data analysis was done by using two-way GLM ANOVA for repeated measures, entering site as a random factor; treatment was black vs. transparent Perspex.

We restricted further analysis only to trials done with black Perspex. We calculated the same ratios, this time for 5-min intervals and associated them to mean light intensity measured during the sampled interval. We tested whether reduced ambient light would correspond to a higher numbers of passes (or bats) at the covered watering troughs, as predicted if bats are deterred by the colour of the surface approached. Because the distribution of light intensity measurements did not meet the assumptions of a parametric Analysis of Covariance – ANCOVA [21], we applied Quade’s non-parametric alternative procedure [22].

### Echolocation Differences during Approach

To determine whether approaching water or Perspex induced any difference in echolocation behaviour, we selected *B. barbastellus* as a model species because as illustrated above we were always fully sure of the identity of recordings.

For 30 approaches (15 for either condition, Perspex or water) we could select sufficient echolocation sequences in situations only differing for surface type, all other variables being equal (i.e. bats approaching the same watering trough from the same direction and under a similar angle of attack). We measured the interpulse interval (IPI, i.e. the time between two consecutive calls) and plotted it vs. time to distinguish between search and approach phases [20] and to identify potential terminal phase events (see “Results”). We used all interpulse intervals corresponding to each phase to determine differences between 1) terminal phase and the remaining approach phase and 2) substrate (water vs. Perspex) by a two-way GLM ANOVA. To avoid pseudo-replication, values were averaged for each sequence and means used for analysis. Data were log-transformed to meet ANOVA assumptions and normality was tested with a Ryan-Joiner test.

We measured duration, starting frequency and terminal frequency from six calls per sequence (three from approach, three from the terminal phase) taken at random from those showing a good signal-to-noise ratio. Duration was measured from oscillograms. We obtained spectrograms with 1024-point FFTs and an FFT Hamming window with a 95% overlap, providing a frequency resolution of 488 Hz. We measured the frequency of maximum energy (FMAXE), the starting (SF) and end (EF) frequencies from power spectra. SF and EF were measured at –25 dB relative to the amplitude of the frequency of maximum energy from the corresponding power spectra [23], [24]. In this way we reduced subjectivity and ensured consistency in measurements – e.g. [24]. For search phase calls we refer to call types1 and 2 following description provided in the scientific literature [18], [20], [25].

We used mean values obtained from the six calls per sequence to determine differences between phase (approach vs. terminal) and substrate (water vs. Perspex) by a two-way GLM ANOVA.

Statistical tests were carried out with Minitab rel. 13. In all tests, significance was set at P<0.05.

## Results

### Experiment 1: Water vs. Transparent Perspex

**Figure 1:**
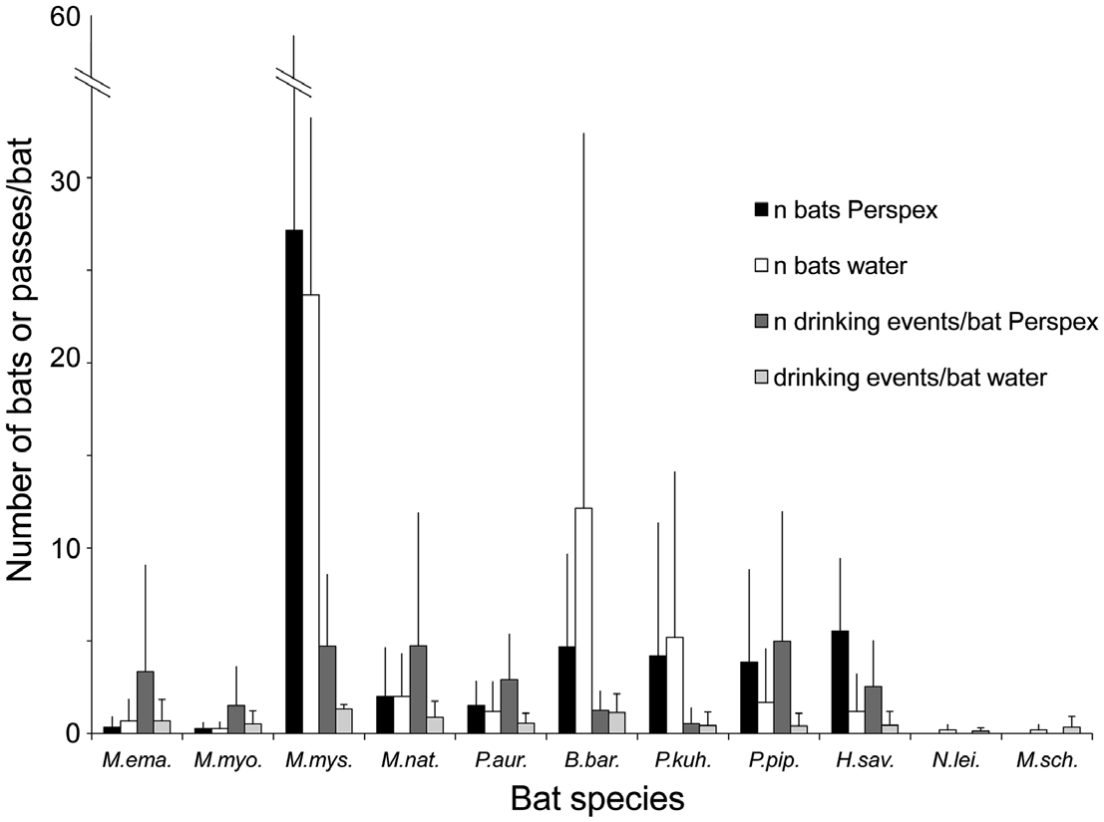
Mean numbers of bats and mean numbers of drinking attempts per bat on Perspex vs. water categorized by species. Error bars show the standard deviation. *M. ema.*  =  *Myotis emarginatus*, *M.myo*  =  *Myotis myotis*, *M.mys.*  =  *Myotis mystacinus M.nat.*  =  *Myotis nattereri*, *P.aur.*  =  *Plecotus auritus*, *B.bar.*  =  *Barbastella barbastellus*, *P.kuh.*  =  *Pipistrellus kuhlii*, *P.pip.*  =  *Pipistrellus pipistrellus*, *H.sav.*  =  *Hypsugo savii*, *N.lei.*  =  *Nyctalus leisleri*, *M.sch.*  =  *Miniopterus schreibersii.*

In the first experiment we recorded 1484 drinking attempts by 299 bats on Perspex and 407 drinking events by 287 bats on water from 11 species (*M. emarginatus*, *M. myotis*, *M. mystacinus*, *M. nattereri*, *P. auritus*, *B. barbastellus*, *M. schreibersii*, *N. leisleri*, *P. kuhlii*, *P. pipistrellus*, *H. savii*; Figure 1). The most frequently recorded bats were *M. mystacinus* and *B. barbastellus*; some species were only occasionally observed (Figure 1). Only three species (*M. mystacinus*, *B. barbastellus* and *P. auritus*) were recorded at all sites. The maximum number of drinking attempts made by an individual on Perspex largely varied both within and across species, from 1 up to 44 (as seen in one *H. savii*) or 45 (one *M. mystacinus*), but in most cases this was <10. On average, a single bat attempted to drink 5.4±5.6 times from Perspex vs. 1.4±0.6 times from water (ANOVA, F_1,49_ = 13.35, P<0.005; Figure 1) until it either gave up or moved to drink at the uncovered watering trough. When a bat repeatedly attempted to drink from Perspex, it did so trying again approximately every 2–3 sec, so its behaviour was conspicuous and tracking it from audio and video recordings generally obvious. Bats approaching Perspex actually touched the surface as they did to drink from the uncovered watering trough.

The overall number of approaches (log-transformed values) was only influenced by which watering trough was covered with Perspex during the experiment but did not differ between Perspex and water or across sites (Table 1). The same result was obtained for the number of bats approaching Perspex vs. water. The GLM ANOVA done on the number of drinking attempts per bat carried out on Perspex vs. water provided a different result: a significantly larger number of attempts were made on Perspex than on water (Table 1; Figure 1) but neither which watering trough was covered during the experiment nor the site influenced this variable.

**Table 1:** Results of repeated measures General Linear Model ANOVA for the effect of watering trough covered, substrate type – water or Perspex – and site, on: a) the total number of drinking approaches to either surface (Perspex vs. water); b) the number of individual bats performing them; and c) the number of approaches per bat.

		d.f.	*F*	*P*
All bats				
Overall number ofapproaches*	Watering trough covered	1,7	22.56	<0.005
	Substrate	1,7	2.87	*n.s.*
	Site	2,7	0.39	*n.s.*
Number of approachingbats	Watering trough covered	1,7	6.83	<0.05
	Substrate	1,7	0.00	*n.s.*
	Site	2,7	2.62	*n.s.*
Number of drinkingattempts/bats	Watering trough covered	1,7	2.12	*n.s.*
	Substrate	1,7	7.16	<0.05
	Site	2,7	0.70	*n.s.*
***Myotis mystacinus***				
Overall number ofapproaches*	Watering trough covered	1,7	18.60	<0.005
	Substrate	1,7	3.69	*n.s.*
	Site	2,7	1.12	*n.s.*
Number of approachingbats*	Watering trough covered	1,7	11.29	<0.05
	Substrate	1,7	0.00	*n.s.*
	Site	2,7	2.62	*n.s.*
Number of drinkingattempts/bats	Watering trough covered	1,7	1.64	*n.s.*
	Substrate	1,7	6.16	<0.05
	Site	2,7	0.70	*n.s.*
***Barbastella barbastellus***				
Overall number ofapproaches	Watering trough covered	1,6	1.85	*n.s.*
	Substrate	1,6	0.39	*n.s.*
	Site	2,6	1.52	*n.s.*
Number of approachingbats	Watering trough covered	1,6	2.33	*n.s.*
	Substrate	1,6	1.40	*n.s.*
	Site	2,6	1.32	*n.s.*
Number of drinkingattempts/bats	Watering trough covered	1,6	2.33	*n.s.*
	Substrate	1,6	1.40	*n.s.*
	Site	2,6	1.32	*n.s.*
***Plecotus auritus***				
Overall number ofapproaches*	Watering trough covered	1,8	2.99	*n.s.*
	Substrate	1,8	8.01	0.047
	Site	2,8	10.28	<0.05
Number of approachingbats	Watering trough covered	1,8	14.38	<0.05
	Substrate	1.8	0.01	*n.s.*
	Site	2,8	9.58	<0.05
Number of drinkingattempts/bats	Watering trough covered	1,8	0.19	*n.s.*
	Substrate	1,8	9.03	<0.05
	Site	2,8	2.31	*n.s.*

Site was entered as a random factor. No interaction was significant so these were removed from final models. (*)  =  data log-transformed to meet the ANOVA assumptions; d.f.  =  degree of freedom.

We repeated this analysis only for the species that occurred at all sites during the experiment. For *M. mystacinus* we obtained the same outcome of the analysis carried out on all bats (Table 1).

The total number of *B. barbastellus* approaches was not influenced by surface type, watering trough covered during the trial or site (Table 1). The same results were obtained for the number of *B. barbastellus* bats approaching Perspex vs. water as well as for the number of drinking attempts per bat (Table 1). *P. auritus* made more drinking attempts on Perspex (10.2±11.1) than on water (1.6±2.51) yet the test’s significance value was borderline (Table 1). Site, but not watering trough covered had a significant effect on this variable. Both site and watering trough covered influenced significantly the number of bats attempting to drink, but surface type had no effect (Table 1). The number of drinking attempts per bat was significantly higher at watering troughs covered with Perspex (4.3±4.0) than at those (0.6±0.6) left uncovered but neither site nor the watering trough covered influenced this variable (Table 1).

**Figure 2:**
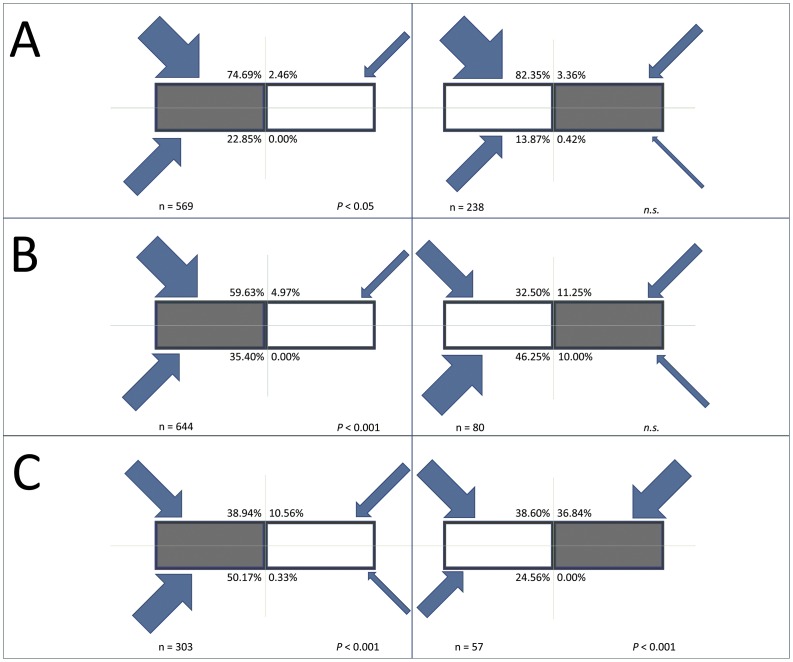
Schematic bird’s eye view of watering troughs manipulated for the experiment at sites A, B and C. For each site one watering trough was covered with Perspex (grey rectangle), the other was left uncovered (white) so that water was available to bats. Two replicates of the experiment were done at each site, covering a different watering trough each time (left and right respectively). The arrows indicate the general direction (±45°) from which the bats approached the watering trough for drinking. Arrow width is proportional to the numbers of bats approaching from each direction (percent values are also given). Most bats always came from one side (and, in four out of six cases, one quadrant – see *P* values of Fisher’s exact tests in figure) and drank at the first watering trough encountered, so that the latter was disproportionately used over the other regardless of whether it had been covered with Perspex or not.

The above analyses illustrated that drinking bats tended to prefer one watering trough over another in all trials. An assessment of the directions followed by bats to reach the watering troughs in the six trials showed that most bats always came from one side (and, in four out of six cases, one quadrant was significantly selected over the others – see Fisher’s exact tests in Figure 2) and drank at the first watering trough encountered, so that the first watering trough was disproportionately used over the other regardless of whether it had been covered with Perspex or not (Figure 2).

### Experiment 2: Water vs. Black Perspex

Bats equally mistook transparent or black Perspex for water. Neither Perspex type (transparent vs. black Perspex) nor site influenced significantly the ratio between the number of approaches to either surface type and the overall (Perspex + water) number of approaches recorded (transparent vs. black Perspex: F_2,5_ = 1.27, *n.s.*; site: F_2,5_ = 0.91, *n.s.*). An identical result was obtained for the “number of bats approaching Perspex/overall number of bats” ratio (transparent vs. black Perspex: F_2,5_ = 14.1, *n.s.*; site: F_2,5_ = 6.03, *n.s.*). The analysis at species level was only performed for *M. mystacinus* because this was the only bat for which sufficient numbers of subjects were recorded at drinking sites (both at covered and uncovered watering troughs) during the experiment done with black Perspex. In this case no significant effect was detected: for the number of approaches ratio, transparent vs. black Perspex: F_2,5_ = 5.85, *n.s.*; site: F_2,5_ = 3.73, *n.s.*; for the number of bats ratio, transparent vs. black Perspex: F_2,7_ = 3.54, *n.s.*; site: F_2,5_ = 5.52, *n.s.*

We then tested whether light or site influenced the number of approaches made by bats on black Perspex divided by the overall approach number (black Perspex + water) within 5-min intervals. During the experiment, mean light intensity (calculated from the 5-min interval values) at the three sites was 1.2±1.1 lux, 0.5±0.3 lux, and 0.2±0.1 lux, lux respectively, whereas the corresponding fraction of the moon illuminated on those nights (data US Naval Observatory, Washington) was 76%, 90%, and 59%. A non-parametric ANCOVA applied to the whole dataset (all species) revealed a positive effect of light (F_1,17_ = 8.69, P<0.01) but no effect of site (F_2,17_ = 2.87, *n.s.*); the ratio between the number of bats approaching Perspex and the total (Perspex + water) showed again a positive effect of light (F_1,17_ = 4.88, P<0.05) and this time also a site effect (F_2,17_ = 6.12, P<0.05). In summary, bats apparently mistook Perspex for water even more frequently when more light was available, i.e. when colour should have been more easily detected.

The same analysis done on the *M. mystacinus* dataset failed to detect significant differences (number of approaches to black Perspex/overall approach number within 5-min intervals: light, F_1,17_ = 0.33, *n.s.*; site, F_2,17_ = 2.56, *n.s.*; number of bats approaching black Perspex/overall number of bats within 5-min intervals: light, F_1,17_ = 0.16, *n.s.*; site, F_2,17_ = 3.34, *n.s.*).

### Echolocation Differences during Approach to Perspex vs. Water in B. barbastellus

When approaching water or Perspex, *B. barbastellus* broadcast similar echolocation sequences, only made of calls represented by frequency modulated sweeps. Before initiating the approach (search phase), both call types 1 and 2 were alternated. In the approach phase, calls resembled modified type 2 calls, showing a steeper frequency modulated shape and a broader bandwidth (Table 2). Plotting IPI vs. time we recognized the approach phase characterised by a decreasing IPI trend, and a terminal phase during which IPI did not decrease further (a typical echolocation approach sequence of a drinking *B. barbastellus* is shown in Figure S1). The terminal phase consisted of groups of two or more calls. Within such groups, IPIs measured ca. 10 ms, whereas consecutive groups were spaced out by longer IPIs (ca. 20 ms). For the 30 sequences selected, we obtained mean values/sequence from 483 approach phase (Perspex = 259; water = 224) and 529 terminal phase (Perspex = 238; water = 291) IPIs respectively. IPI did differ between phases but not between substrate types (Table 2). We found significant differences between echolocation calls in the approach and the terminal phase respectively. Compared with the echolocation pulses in the approach phase, those in the terminal phase had lower FMAXE and EF and a shorter duration, but SF did not differ significantly between phases (Table 2). No significant difference was detected between calls emitted by bats approaching water vs. Perspex, i.e. the two surfaces elicited identical echolocation behaviour.

**Table 2:** Descriptive statistics and results of General Linear Model (GLM) Analysis of Variance for echolocation calls of approach and terminal phases recorded from *B. barbastellus* approaching water or Perspex.

**Interpulse interval (ms)**	Phase	*N*	Substrate (SD)	
			Water	Perspex
	Approach	30	34.1 (8.9)	31.0 (4.5)
	Terminal phase	30	13.6 (2.1)	12.0 (2.2)
GLM ANOVA (*)				
Factor	d.f.	Adj. MS	F	P
Phase	1	0.0265	325.44	< 0.001
Substrate	1	2.419	3.57	*n.s.*
Phase x Substrate	1	0.002	0.29	*n.s.*
Error	56	0.007		
**Duration (ms)**	Phase	*N*	Substrate (SD)	
			Water	Perspex
	Approach	30	2.3 (0.3)	1.9 (0.4)
	Terminal phase	30	1.2 (1.0)	1.2 (0.9)
GLM ANOVA				
Factor	d.f.	Adj. MS	F	P
Phase	1	11.88	122.31	< 0.001
Substrate	1	0.37	3.79	*n.s.*
Phase x Substrate	1	0.40	4.12	*n.s.*
Error	56			
**FMAXE (kHz)**	Phase	*N*	Substrate (SD)	
			Water	Perspex
	Approach	30	41.1 (1.4)	41.3 (2.1)
	Terminal phase	30	37.1 (2.7)	38.0 (3.6)
GLM ANOVA				
Factor	d.f.	Adj. MS	F	P
Phase	1	204.98	30.76	< 0.001
Substrate	1	4.76	0.71	*n.s.*
Phase x Substrate	1	1.63	0.25	*n.s.*
Error	56			
**SF (kHz)**	Phase	*N*	Substrate (SD)	
			Water	Perspex
	Approach	30	48.8 (2.0)	49.5 (1.3)
	Terminal phase	30	49.2 (2.0)	49.6 (1.6)
GLM ANOVA				
Factor	d.f.	Adj. MS	F	P
Phase	1	0.94	0.31	*n.s.*
Substrate	1	5.46	1.80	*n.s.*
Phase x Substrate	1	0.28	0.1	*n.s.*
Error	56			
**EF (kHz)**	Phase	*N*	Substrate (SD)	
			Water	Perspex
	Approach	30	31.0 (1.7)	31.1 (2.6)
	Terminal phase	30	27.1 (1.2)	27.4 (1.5)
GLM ANOVA				
Factor	d.f.	Adj. MS	F	P
Phase	1	214.70	65.30	< 0.001
Substrate	1	0.58	0.18	*n.s.*
Phase x Substrate	1	0.28	0.09	*n.s.*
Error	56			

(*)  =  analysis done on log-transformed data. FMAXE  =  Frequency of Maximum Energy; SF, EF  =  Starting and Terminal Frequencies taken at -25 dB below the frequency of maximum energy. Interactions between factors are indicated with a ‘x’ sign. SD  =  standard deviation, d.f.  =  degrees of freedom; Adj. MS  =  adjusted mean squares; n.s.  =  not significant (P > 0.05).

## Discussion

### Bats Mistake Any Horizontal Smooth Surface for Water in Field Tests

We confirmed that, as in laboratory trials [14], under natural conditions too bats may be deceived by a smooth horizontal surface and exhibit repeated drinking attempts. We are confident that our results reveal a general pattern which we verified for 11 species and many individuals. Echoacoustic cues provided by the water-like Perspex surface clearly dominated over all other information. When bats where presented with an equal surface of Perspex and true water, neither the number of total approaches across species nor that for individual species of *M. mystacinus* and *B. barbastellus* differed between the two surface types. *P. auritus* even showed a greater number of approaches to Perspex.

As we showed for *B. barbastellus*, water and Perspex surfaces elicited similar echolocation behaviour, further supporting that bats approaching Perspex tried to drink – as verified by video recordings – rather than to simply explore the surface, i.e. they were unable to distinguish between real water and an artificial smooth surface. In no case did the approaches made by bats represent attempts to capture prey resting on the Perspex layer: the terminal phase we recorded clearly differed from feeding buzzes (fast repetitions of brief calls emitted by bats close to prey; Kalko and Schnitzler 1989) which in foraging *B. barbastellus* show shorter IPIs and are made of two distinct phases, buzz I and buzz II [20], [26].

Although we did not analyze them, a terminal phase distinct from a typical feeding buzz was also noticed in the other species recorded (*pers. obs.*) and is probably a generalized pattern used to guide a safe drinking manoeuvre.

In situations where many bats fly together such as a drinking site visited by hundreds to thousands of individuals per night, it can be argued that echo-acoustic detection of obstacles can be confused by the many calls that are broadcast (and the corresponding echoes), so other cues, or spatial memory, could play an important role [27], [28]. However, bats have been found to strictly rely on echoacoustic cues even when flying at familiar sites such as roosts, typically in groups of conspecifics [29] and may still effectively avoid objects placed along their route [28].

For water recognition echolocation is so important because of its dual function, i.e. assessing target properties such as density and texture [30], as well as obtaining crucial information to orientate and determine distance to surrounding objects [31]. When approaching water, echolocation is clearly used to recognize the water surface but also to precisely evaluate the distance to water and ensure a safe drinking manoeuvre: any misjudgement would mean to crash into the water and thus expose the bat to heat loss and also increase the risks of predation and injury.

In our experiment, vision had no discernible effect on water recognition, i.e. bats equally mistook transparent or black Perspex for water. Moreover, when the dataset including all species was considered and the effect of light taken into account, bats apparently mistook Perspex for water even more frequently under higher ambient light intensity, contrary to what expected if vision was involved and colour had a deterring role. However, we believe that in this case it is unlikely that the coloured surface was inherently more attractive for bats. We can only speculate that under brighter ambient light vision may have favoured a process of social imitation which has resulted in a concentration of bats over the same watering trough – the one covered with Perspex, where bat activity was more conspicuous because of the repeated drinking attempts occurring there. Visually detecting other drinking bats, along with eavesdropping on their echolocation calls [32] might speed up the process of locating new water sources.

Water colour may be influenced by several factors (some of which may change over time) e.g. water depth, presence of suspended particles, type of substrate, seasonal presence of aquatic plants, colonization by benthic organisms. Colour is also only detectable under sufficient light and thus for a limited time (i.e. around emergence time or on full moon nights). These points would suggest a secondary role of vision for water recognition, all the more in a natural setting.

We are aware that the limited time during which brighter light was available in our test – corresponding to the first half of each trial or less – may have been insufficient to detect any influence of vision, but in designing our experiment we avoided manipulating ambient light levels because we aimed to avoid disrupting natural behaviour patterns.

*Myotis lucifugus* [33] was observed to collide against stationary objects more often in the light, a fact suggesting that bats relied on vision besides echolocation in the presence of visual cues, but their limited visual capabilities at higher illumination levels resulted in a higher collision rate. Small *Myotis* bats such as *M. lucifugus*, and probably *M. mystacinus* which was the dominant species in our samples, have poor visual acuity compared to other bats [34], [35] and this may have determined the lack of reaction to visual cues. In laboratory tests it was noticed [14] that rates of drinking attempts at an artificial surface mimicking water dropped when trials took place under lit rather than unlit conditions. However, this was tested on *M. schreibersii*, a species only occasionally present during our trials. For some bat species, vision plays an important role in hunting too [36], [37]. The role of colour for water recognition might be more conspicuous in species relying on vision to forage. Although our sample featured one of them, i.e. *P. auritus* [36], the number of drinking attempts we recorded from it in experiment 2 was too limited to detect any effect. Further experiments should explore the existence of interspecific differences in the contribution of vision to water recognition and also test whether surfaces of brighter colours would be more conspicuous to bats.

As far as other sensorial cues (particularly, olfactory or tactile ones) are concerned, our results support the hypothesis that they are negligible as suggested in laboratory studies [14]. We reach this conclusion because if other cues were important for water recognition, the most common reaction of bats to Perspex would have been to immediately refrain from attempting to drink further after the very first approach, which involves a close-range assessment of the substrate.

### A Role for Learning Effects

One of our objectives was to explore whether under natural conditions (where bats may decide to leave to reach another drinking site) an unsuccessful drinking experience as that determined by the artificial surface would override the influence of the acoustic water-like cues the latter provided. We have seen that in several cases the Perspex surface deceived a bat so effectively that, as seen in laboratory trials [14], many consecutive drinking attempts were made before giving up. However, in most cases bats lost motivation after less than 10 attempts and either disappeared from the scene or moved to the uncovered water surface where they drank successfully. Our experimental design was especially suited to test this because uncovered water was available at all sites. In the laboratory experiments [14] bats were presented with a smooth horizontal surface mimicking water and real water was offered only after removing the former. Under such conditions bats showed high rates of drinking attempts at the fake water surface, sometimes 100 or more.

In our experiments, when bats left the watering trough covered with Perspex and moved to the control watering trough they made a marked change in the flight path and the approaching manoeuvre, i.e. they did not appear to randomly sample all potentially available water surfaces until real water was finally located. Based on the unsuccessful drinking attempts, bats must have modified their behaviour as soon as the experience gathered has overridden the influence of echoacoustic information. We do not know whether some of the bats leaving the site may have returned later to try and drink again from Perspex (in this case, the two events would have been recorded separately and attributed to different subjects). Overall, although acoustic recognition itself is stereotyped and its importance in the process overwhelming, our findings suggest that experience plays a role to increase behavioural flexibility. The adaptive value of this flexibility is clear: for example, it allows a bat encountering a frozen water surface or a human-made horizontal artificial surface to save energy by modifying its behaviour to avoid being deceived for too long.

### Effects of Spatial Memory and Social Interactions

On a larger scale, involving site recognition rather than water detection, our experiment pointed at a role for spatial memory. In fact, we found that at all experimental sites one of the two watering troughs received a disproportionately higher number of drinking attempts (as seen from the analysis of the whole dataset and *M. mystacinus*) and was visited by more bats (as found for the whole dataset, *M. mystacinus* and *P. auritus*), regardless of whether it was covered with Perspex or not. It is important to notice that most bats followed the same general route to reach the drinking site, so that the “preferred” drinking watering trough was the one first encountered by approaching bats. This result agrees with the findings presented in a study [38] which explained the adoption of such unidirectional flight paths in terms of cooperation aimed at reducing the risk of collision between bats. Both spatial memory and social interactions may play important roles in determining such preferentially used routes.

## Supporting Information

Figure S1 **Echolocation sequence of a drinking barbastelle bat (** ***Barbastella barbastellus*** **).** (a) spectrogram showing the approach and the terminal phases. The red arrow shows the noise produced when the bat makes contact with the water, which was clearly audible in many recordings made over water. (b) Interpulse interval (IPI) plotted vs. time of the same sequence. Note how the terminal phase is made of groups of calls broadcast with a high pulse rate separated by longer IPIs.
(TIF)

## References

[1] Schmidt-Nielsen K (1997) Animal physiology. Adaptation and environment. Cambridge: Cambridge University Press. 607 p.

[2] Chew RM, White HE (1960) Evaporative water losses of the pallid bat. J Mammal 41: 452–458.

[3] Thomas DW, Cloutier D (1992) Evaporative water loss by hibernating little brown bats, *Myotis lucifugus* . Physiol Zool 65: 443–456.

[4] Muñoz-Garcia A, Ro J, Reichard JD, Kunz TH, Williams JB (2012) Cutaneous water loss and lipids of the stratum corneum in two syntopic species of bats. Comp Biochem Physiol A Mol Integr Physiol 161: 208–15. 22079104 10.1016/j.cbpa.2011.10.025

[5] Speakman JR, Racey PA (1989) Hibernal ecology of the pipistrelle bat: energy expenditure, water requirements and mass loss, implications for survival and the function of winter emergence flights. J Anim Ecol 58: 797–813.

[6] Boyles JG, Dunbar MB, Whitaker JO Jr (2006) Activity following arousal in winter in North American vespertilionid bats. Mamm Rev 36: 267–280.

[7] Adams RA, Hayes MA (2008) Water availability and successful lactation by bats as related to climate change in arid regions of western North America. J Anim Ecol 77: 1115–1121. 18684132 10.1111/j.1365-2656.2008.01447.x

[8] Rainho A, Palmeirim JM (2011) The Importance of Distance to Resources in the Spatial Modelling of Bat Foraging Habitat. PLoS ONE 6(4): e19227 doi:10.1371/journal.pone.0019227. 21547076 10.1371/journal.pone.0019227 PMC3081845

[9] Kwiecinski GG, Krook L, Wimsatt WA (1987) Annual skeletal changes in the little brown bat, *Myotis lucifugus lucifugus*, with particular reference to pregnancy and lactation. Am J Anat 178: 410–420. 3604957 10.1002/aja.1001780410

[10] Barclay RMR (1994) Constraints on reproduction by flying vertebrates-energy and calcium. Am Nat 144: 1021–1031.

[11] Bernard RTF, Davison A (1996) Does calcium constrain reproductive activity in insectivorous bats? Some empirical evidence for Schreibers’ long-fingered bat (*Miniopterus schreibersii*). S Afr J Zool 31: 218–220.

[12] Bravo A, Harmsa KE, Emmons LH (2010) Puddles created by geophagous mammals are potential mineral sources for frugivorous bats (Stenodermatinae) in the Peruvian Amazon. J Trop Ecol 26: 173–184.

[13] Voigt CC, Capps KA, Dechmann DKN, Michener RH, Kunz TH (2008) Nutrition or detoxification: why bats visit mineral licks of the Amazonian rainforest. PLoS ONE 3(4): e2011 doi:10.1371/journal.pone.0002011. 18431492 10.1371/journal.pone.0002011 PMC2292638

[14] Greif S, Siemers BM (2010) Innate recognition of water bodies in echolocating bats. Nature Communications, doi: 10.1038/ncomms1110. 10.1038/ncomms1110 PMC3060641 21045825

[15] Siemers BM, Page RA (2009) Behavioral studies of bats in captivity: Methodology, training, and experimental design. In: Kunz TH, Parsons S, editors. Ecological and behavioral methods for the study of bats. 2nd edition, Baltimore: Johns Hopkins University Press. 373–392.

[16] Wolff JO (2003) Laboratory studies with rodents: facts or artifacts? Bioscience 53: 421–427.

[17] Eklöf J (2003) Vision in echolocating bats. Doctoral thesis Zoology Department, Göteborg University.

[18] Russo D, Jones G (2002) Identification of twenty–two bat species (Mammalia: Chiroptera) from Italy by analysis of time-expanded recordings of echolocation calls. J Zool (Lond) 258: 91–103.

[19] Vaughan N, Jones G, Harris S (1997) Identification of British bat species by multivariate analysis of echolocation call parameters. Bioacoustics 7: 189–207.

[20] Denzinger A, Siemers BM, Schaub A, Schnitzler HU (2001) Echolocation by the barbastelle bat, *Barbastella barbastellus* . J Comp Physiol A 187: 521–528. 11730299 10.1007/s003590100223

[21] Huitema BE (1980) The analysis of covariance and its alternatives. New York: Wiley, 445 p.

[22] Quade D (1967) Nonparametric Analysis of Covariance by Matching. Biometrics 38: 597–611. 7171690

[23] Surlykke A, Moss CF (2000) Echolocation behavior of big brown bats, *Eptesicus fuscus*, in the field and the laboratory. J Acoust Soc Am 108: 2419–2429. 11108382 10.1121/1.1315295

[24] Siemers BM, Schnitzler HU (2004) Echolocation signals reflect niche differentiation in five sympatric congeneric bat species. Nature 429: 657–661. 15190352 10.1038/nature02547

[25] Goerlitz HR, ter Hofstede HM, Zeale MRK, Jones G, Holderied MW (2010) An aerial-hawking bat uses stealth echolocation to counter moth hearing. Curr Biol 20: 1568–1572. 20727755 10.1016/j.cub.2010.07.046

[26] Kalko EKV, Schnitzler H-U (1989) The echolocation and hunting behavior of Daubenton’s bat, *Myotis daubentoni* . Behav Ecol Sociobiol 24: 225–238.

[27] Griffin DR, Webster FA, Michael CR (1960) The echolocation of flying insects by bats. Anim. Behav 8: 141–154.

[28] Goerlitz HR, Genzel D, Wiegrebe L (2012) Bats’ avoidance of real and virtual objects: implications for the sonar coding of object size. Behav Processes 89: 61–67. 22085788 10.1016/j.beproc.2011.10.018

[29] Holland RA, Waters DA (2007) The effect of familiarity on echolocation in the Megachiropteran bat *Rousettus aegyptiacus* . Behaviour 144: 1053–1064.

[30] Schmidt S (1988) Evidence for a spectral basis of texture perception in bat sonar. Nature 331: 617–619. 3340212 10.1038/331617a0

[31] Neuweiler G (1988) Foraging ecology and audition in echolocating bats. Trends Ecol Evol 4: 160–166. 10.1016/0169-5347(89)90120-1 21227342

[32] Fenton MB (2003) Eavesdropping on the echolocation and social calls of bats. Mamm Rev 33: 193–204.

[33] Orbach DN, Fenton B (2010) Vision impairs the abilities of bats to avoid colliding with stationary obstacles. PLoS ONE 5(11): e13912 doi:10.1371/journal.pone.0013912. 21085481 10.1371/journal.pone.0013912 PMC2976695

[34] Suthers RA (1966) Optomotor responses by echolocating bats. Science 152: 1102–1104. 5931464 10.1126/science.152.3725.1102

[35] Bell GP, Fenton MB (1986) Visual acuity, sensitivity and binocularity in a gleaning insectivorous bat, *Macrotus californicus* (Chiroptera: Phyllostomidae). Anim Behav 34: 409–414.

[36] Eklöf J, Jones G (2003) Use of vision in prey detection by brown long-eared bats, *Plecotus auritus* . Anim Behav 66: 949–953.

[37] Rydell J, Eklöf J (2003) Vision complements echolocation in an aerial-hawking bat. Naturwissenschaften 90: 481–483. 14564410 10.1007/s00114-003-0464-x

[38] Adams RA, Simmons JA (2002) Directionality of drinking passes by bats at water holes: is there cooperation? Acta Chiropt 4: 1–5.

