# Supplementary figures and images for: Sensory Ecology of Water Detection by Bats: A Field Experiment
Source: PLoS One. 2012 Oct 25;7(10):e48144. doi: 10.1371/journal.pone.0048144 (PMC3483877; doi:10.1371/journal.pone.0048144)

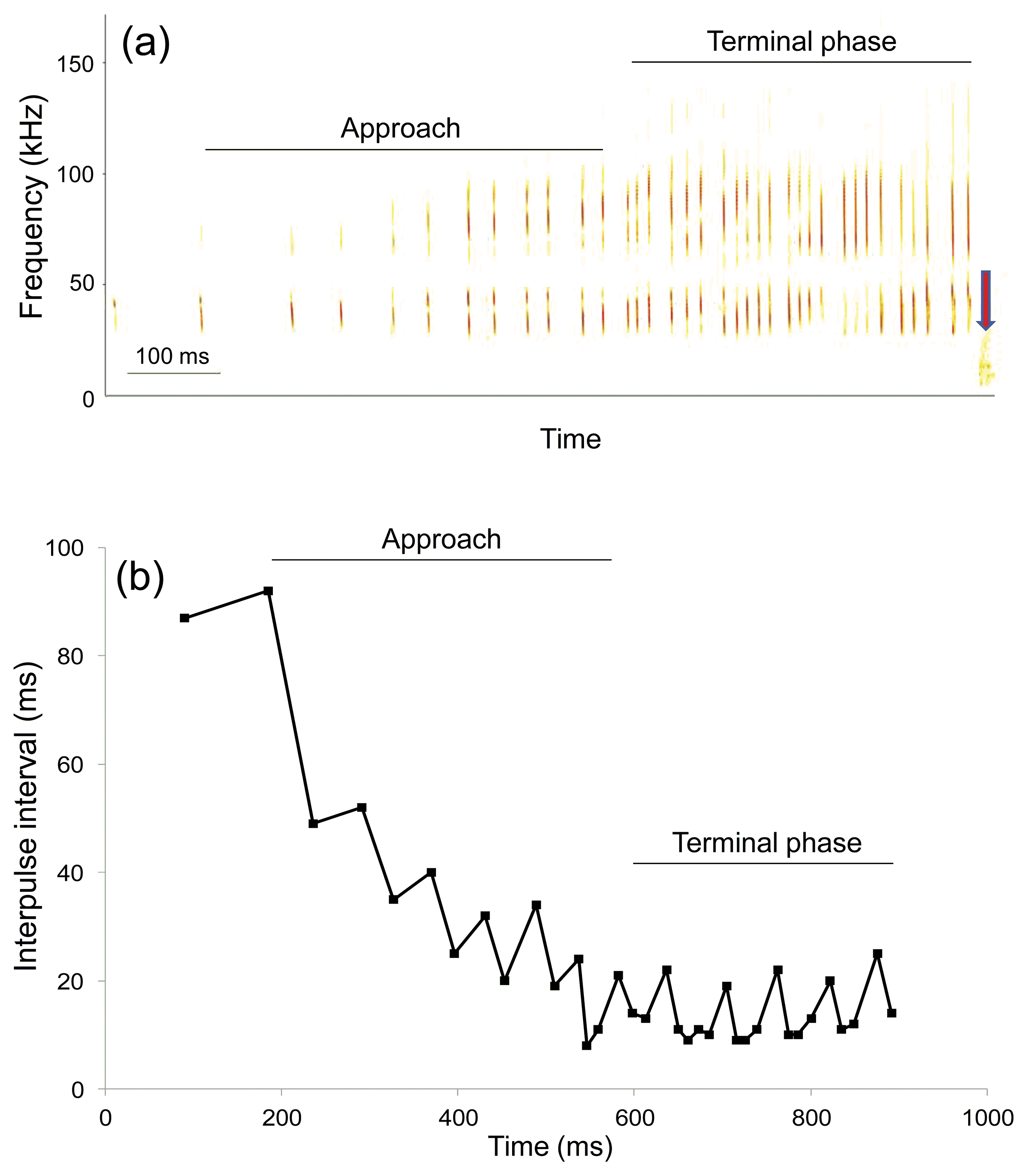

Supplement: Figure S1 — Echolocation sequence of a drinking barbastelle bat ( Barbastella barbastellus ). (a) spectrogram showing the approach and the terminal phases. The red arrow shows the noise produced when the bat makes contact with the water, which was clearly audible in many recordings made over water. (b) Interpulse interval (IPI) plotted vs. time of the same sequence. Note how the terminal phase is made of groups of calls broadcast with a high pulse rate separated by longer IPIs. (TIF) [file pone.0048144.s001.tif]
